# Prognostic value of brain natriuretic peptide and N-terminal pro b-type natriuretic peptide in patients with cardiac arrest: A systematic review and meta-analysis

**DOI:** 10.12669/pjms.42.4.14657

**Published:** 2026-04

**Authors:** Yan Zhang, Jiacheng Wu, Yunxia Qin, Zhenzhen Zhang

**Affiliations:** 1 Yan Zhang Department of Emergency, Department of Emergency, Nantong Tumour Hospital & Affiliated Tumour Hospital of Nantong University, Nantong, Jiangsu Province 226001, P.R. China; 2 Jiacheng Wu Department of Urology, Department of Emergency, Nantong Tumour Hospital & Affiliated Tumour Hospital of Nantong University, Nantong, Jiangsu Province 226001, P.R. China; 3 Yunxia Qin Department of Surgery, Department of Emergency, Nantong Tumour Hospital & Affiliated Tumour Hospital of Nantong University, Nantong, Jiangsu Province 226001, P.R. China; 4 Zhenzhen Zhang Department of Emergency, Department of Emergency, Nantong Tumour Hospital & Affiliated Tumour Hospital of Nantong University, Nantong, Jiangsu Province 226001, P.R. China

**Keywords:** Cardiopulmonary arrest, Mortality, Biomarker, Functional outcome

## Abstract

**Objective:**

The aim of the present study was to systematically review published evidence on the prognostic ability of brain natriuretic peptide (BNP) and N-terminal pro b-type natriuretic peptide (NT-proBNP) in patients with cardiac arrest.

**Methodology:**

This review searched PubMed, Embase, Scopus, and Cochrane databases for studies assessing mortality, survival to discharge, return of spontaneous circulation (ROSC), or neurological outcomes in cardiac arrest patients based on BNP or NT-proBNP. Search limits were from database inception to 20^th^ March 2025.

**Results:**

Eleven studies were eligible. Meta-analysis showed a statistically significant association between high baseline BNP levels and mortality after cardiac arrest (OR: 2.47 95% CI: 1.24, 4.92 I^2^=90%). However, the results turned non-significant on exclusion of an outlier study (OR: 1.37 95% CI: 0.91, 2.07 I^2^=73%). Pooled analysis indicated that high baseline BNP levels were predictive of poor neurological outcomes (OR: 2.68 95% CI: 1.19, 6.04 I^2^=61%). This result was also not stable on sensitivity analysis. Association between BNP levels and ROSC and survival to discharge was conflicting. Pooled analysis also showed that NT-proBNP was not a significant predictor of mortality after cardiac arrest (OR: 1.16 95% CI: 0.95, 1.42 I^2^=76%), but descriptive analysis indicated that it could be a predictor of poor neurological outcomes.

**Conclusions:**

Evidence on the prognostic ability of BNP and NT-proBNP for cardiac arrest is preliminary. BNP and NT-proBNP may potentially predict poor neurological outcomes after cardiac arrest. Limited and inconsistent evidence also shows that there may be no association between baseline level of these markers and mortality. Data on ROSC and survival to discharge is scarce and conflicting and needs more studies for robust conclusions. Given the high heterogeneity and lack of stability of outcomes on sensitivity analysis, the results must be interpreted with caution.

***Registration No:*** PROSPERO (CRD420251016580).

## INTRODUCTION

Cardiac arrest represents a catastrophic clinical event characterized by the sudden cessation of cardiac function, leading to the cessation of systemic circulation and, consequently, the deprivation of oxygen and essential nutrients to vital organs.^1^ The global incidence of out-of-hospital cardiac arrest is 55 per 100,000 person-years, that accounts for 50% of cardiovascular deaths and nearly 10% of global mortality.^2^ Numerous variables, such as age, sex, length of cardiopulmonary resuscitation (CPR), the existence of shockable rhythms, early defibrillation, and the underlying cause, affect the prognosis of cardiac arrest.^1^ Although a significant proportion of patients who suffer cardiac arrest can be initially stabilized hemodynamically, prognosis remains poor with a survival-to-hospital-discharge rate of only 8.8% and 1-month survival of only 10.7%.^3^ Current guidelines advocate a multimodal strategy incorporating clinical assessment, neurophysiological evaluations including electroencephalogram and somatosensory evoked potentials, radiological imaging of the brain, and brain injury biomarkers to predict outcomes of cardiac arrest.^4^,^5^

However, such a detailed investigation can often be time-consuming. There has also been research suggesting that blood-based transcriptome-based signatures like MAPK3, BCL2 and AKT1 can predict cardiac arrest outcomes.^6^ However, routine availability of such markers is questionable. Given the poor prognosis, the identification of robust and readily accessible biomarkers capable of accurately predicting outcomes following cardiac arrest is of paramount importance.^7^ Such biomarkers could prove invaluable in facilitating early risk stratification, guiding therapeutic interventions, and ultimately improving patient outcomes.^8^

Natriuretic peptides, including brain natriuretic peptide (BNP) and N-terminal pro-B-type natriuretic peptide (NT-proBNP), are cardiac hormones secreted by cardiomyocytes following myocardial stretch and volume overload.^9^ Elevated levels of these peptides are indicative of cardiac dysfunction and have been established as prognostic indicators in various cardiovascular conditions, including heart failure, acute coronary syndromes, valvular heart disease, hypertension, and cardiomyopathies.^10^ However, their utility in cardiac arrest remains understudied. In recent times, a large number of studies^11^-^13^ have reported the prognostic ability of natriuretic peptides for cardiac arrest, but with variable results. In the absence of any systematic review in the literature, it is difficult to analyze the quality of evidence and use these biomarkers in clinical practice. We hereby present the first systematic review and meta-analysis examining the ability of BNP and NT-proBNP to predict outcomes after cardiac arrest.

## METHODOLOGY

The Cochrane Handbook for Systematic Reviews and Meta-analyses^14^ and the Preferred Reporting Items for Systematic Reviews and Meta-analyses (PRISMA) guidelines^15^ were followed by the reviewers while designing, analysing, and reporting the review. The review was registered on PROSPERO (CRD420251016580).

### Information sources:

Articles for the review were obtained by a detailed and independent search conducted by two reviewers on the PubMed, Embase, Scopus, and Cochrane (CENTRAL) databases. Search limits were from database inception to 20^th^ March 2025. We searched for only English language studies conducted on humans. Broadly, the keywords, “cardiac arrest, cardiopulmonary arrest, N-terminal pro B-type natriuretic peptide, NT-proBNP, brain natriuretic peptide, and BNP” were combined using Boolean operators ‘AND’ / ‘OR’ to formulate the search query (Supplementary Table 1). Articles were selected from the databases based on a predetermined eligibility criteria.

**Supplementary Table-I:** Search strategy.

***Embase***
1. 'cardiac arrest'/exp OR 'cardiac arrest '
2. 'cardiopulmonary arrest'
3. #1 OR #2
4. 'N-terminal pro B-type natriuretic peptide'
5. 'brain natriuretic peptide'
6. #4 OR #5
7. #3 and #6
***PubMed***
((cardiac arrest) OR (cardiopulmonary arrest)) AND ((((N-terminal pro B-type natriuretic peptide) OR (NT-proBNP)) OR (brain natriuretic peptide)) OR (BNP)).
***Scopus***
(cardiac arrest OR cardiopulmonary arrest) AND (TITLE-ABS-KEY-AUTH (N-terminal pro B-type natriuretic peptide OR NT-proBNP OR BNP OR brain natriuretic peptide).
***CENTRAL***
((cardiac arrest:ti,ab,kw) OR (cardiopulmonary arrest:ti,ab,kw)) AND ((((N-terminal pro B-type natriuretic peptide:ti,ab,kw) OR (NT-proBNP:ti,ab,kw)) OR (brain natriuretic peptide:ti,ab,kw)) OR (BNP):ti,ab,kw).

### Eligibility:

Studies fulfilling the following criteria were eligible:

- Studies conducted on in-hospital or out-of-hospital cardiac arrest patients.
- Studies examining the prognostic ability of baseline BNP or NT-proBNP on cardiac arrest outcomes.
- Studies reported outcomes like mortality, survival to discharge, return of spontaneous circulation (ROSC), or neurological outcomes.
- All study designs were eligible.

### Excluding criteria:

Studies available only as abstracts, studies not reporting desired outcomes, and studies with overlapping data (in such cases the article with the largest sample size was to be selected).

### Screening:

After the search results had been combined and deduplicated in the reference manager software (Endnote, version 21), two reviewers began screening the studies. First, the titles/ abstracts of eligible records were evaluated for relevance using the criteria listed above. Publications that failed to fulfil the review’s objectives were eliminated at this stage. Full-text publications from relevant studies were retrieved for further evaluation before the final list of studies was developed. We also checked the bibliographies of the included studies for any missing articles. Disagreements in inclusion, if any, were addressed in discussion with the third reviewer.

### Data management:

Data extracted from studies included: first author name, year of publication, study design, inclusion criteria, sample size, demographic details, location of cardiac arrest, comorbidities, shockable rhythm, percentage of survivors, ROSC, marker studies, levels of the marker & timing of measurement, outcomes assessed and follow-up. Two authors extracted all data from the studies independently which was later cross-checked by a third author. Outcomes for the analysis included mortality, survival to discharge, ROSC, or neurological outcomes. None were predefined and definitions of individual studies were accepted.

### Risk of bias:

Quality assessment of studies was done using the non-randomized studies (RoBANS) tool.^16^ Each article was examined on the following domains: patient selection, confounding factors, measurement of exposure, blinding of outcome assessment, incomplete outcome data, and selective reporting. Quality assessment was done by two authors and discrepancies were resolved in consultation with the third reviewer.

### Data analysis:

We conducted a detailed descriptive analysis of all reported outcomes and pooled data when three studies reported similar data for a given outcome. Outcomes were pooled as odds ratios (OR) with 95% confidence intervals (CI) using the generic inverse variance function of “Review Manager” (RevMan, version 5.3). Heterogeneity was assessed using both the Cochrane Q test, which provides the statistical significance and the I² statistic, which quantifies the proportion of total variability. An I² value of 0% shows no observed heterogeneity, while values of 25%, 50%, and 75% correspond to low, moderate, and high heterogeneity, respectively. Given the small number of studies, publication bias could not be assessed. Sensitivity analysis was conducted by excluding one study at a time.

## RESULTS

**Fig.1:**
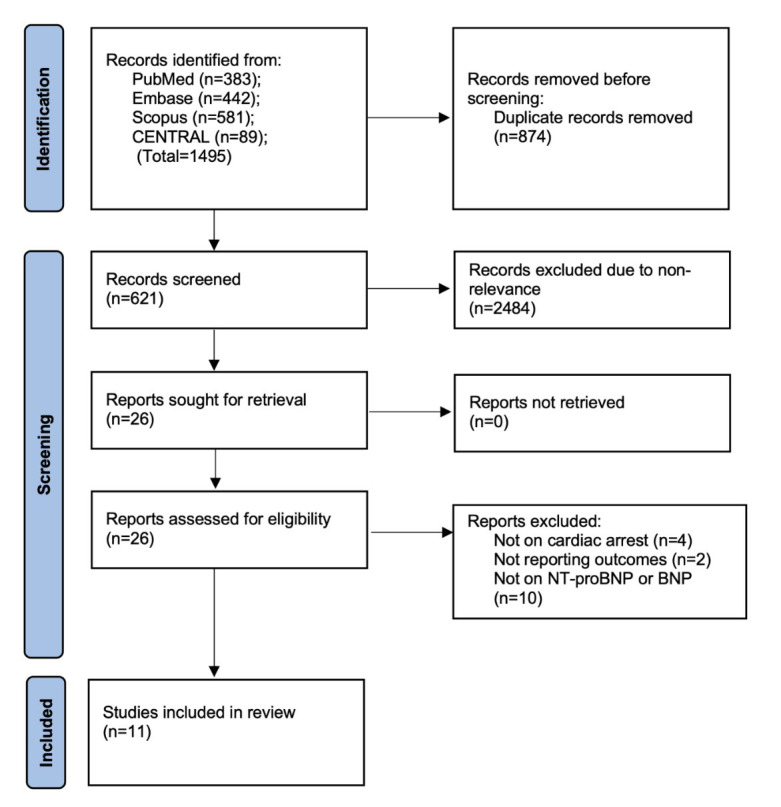
Study flowchart.

We have presented the flowchart of the study as Fig.1. Almost 1,495 articles were obtained from the databases, and after excluding duplicates and primary screening, 26 articles were selected for full-text analysis. After detailed full-text review, 11 studies^11^-^13^,^17^-^24^ were included.

Data extracted from the studies is shown in Table-I. Five studies were retrospective cohort studies, and six were prospective cohort studies. The location of the studies was either Japan, China, Taiwan, India, Korea, Sweden, Norway, Poland, Austria, or the USA. All studies included only adult cardiac arrest patients. In six studies, only those patients with ROSC were included. Seven studies included only out-of-hospital cardiac arrest, while one study included only in-hospital cardiac arrest. The remaining studies included both types of cardiac arrest. Percentage of survivors varied from 3.3 to 80 in the included studies. Five studies reported on BNP, while six studies reported on NT-proBNP. Follow-up of studies ranged from in-hospital to up to one year. Risk of bias analysis is shown in Table-II.

**Table-1:** Details of included studies.

*Study/ Design/ Location*	Inclusion criteria	Sample size	Age (years)	Male gender (%)	Shockable rhythm (%)	Location (%)	DM (%)	HT (%)	HF (%)	CAD (%)	ROSC (%)	Survivors (%)	Marker & timing	Baseline marker level	Outcomes assessed	*F/U*
Jin 2024 R (China)	Adult CA with ROSC	260	63	72.7	82.7	IH 86.2, OH 13.8	24.2	45.4	16.5	11.2	100	80	BNP; peak value during hospitalization	1.008 (0.431, 2.604) ng/mL	Mortality	30-days
Timilsina 2023 P (India)	Adult CA receiving CPR	151	50	64.9	7.9	IH 89.4, OH 10.6	NR	NR	NR	NR	56.9	3.3	NT-proBNP; at resusicitation	NR	ROSC, mortality	7 days
Hong 2023 R (Korea)	Adult OH-CA	732	70	60.3	9	OH 100	NR	NR	5.4	11.2	44.4	8.6	BNP; at resusicitation	NR	ROSC, survival to discharge, neurological outcome	In-hospital
Datta 2021 R (USA)	Adult CA surviving >24 hours	657	63.2	60.4	100	IH 33.8, OH 66.2	43.5	NR	34.3	42.3	100	38.7	BNP; at arrest or post resusicitation	NR	Mortality, neurological outcome	In-hospital
Aarsetoy 2020 P (Norway)	Adult OH-CA	114	67	83	68	OH 100	15	52	26	31	NR	38.6	NT-proBNP; at resusicitation or admission	61 (25, 234) pmol/L	Mortality	30-days
Myhre 2016 P (Norway)	Adult OH-CA with ROSC	155	63	85	100	OH 100	21	45	15	32	100	61.9	NT-proBNP; within 6 hours of CA	706 (213-1624) ng/L	Mortality	1 year
Huang 2016 P (Taiwan)	Adult non-traumatic OH-CA with ROSC	99	70	58.6	14.2	OH 100	30.3	35.3	11.1	25.2	100	45.4	NT-proBNP; after ROSC	NR	Mortality	In-hospital
Frydland 2016 P (Sweden)	Adult comatose OH-CA with ROSC	647	60	80.8	81.3	OH 100	15.5	40.5	6	28	100	53.9	NT-proBNP; after ROSC	NR	Mortality, neurological outcome	6 months
Platek 2015 R (Poland)	Adult IH-CA	106	71.4	57.5	38.7	IH 100	34.9	53.8	23.6	32.1	NR	39.6	NT-proBNP; ICU admission	NR	Mortality	30-days
Sodeck 2007 R (Austria)	Adult comatose OH-CA with ROSC	155	58	69	NR	OH 100	16	28	17	42	100	49	BNP; ED admission	NR	Mortality, neurological outcome	6 months
Nagao 2004 P (Japan)	Adult OH-CA	401	64	80.2	38.5	OH 100	NR	NR	NR	NR	36.5	12.9	BNP; ED arrival	NR	ROSC, mortality, survival to discharge, neurological outcome	In-hospital

CA, cardiac arrest; ROSC, return of spontaneous circulation; IH, in-hospital; OH, out-hospital; DM, diabetes mellitus; HT, hypertension; HF, heart failure; CAD, coronary artery disease; BNP, brain natriuretic peptide; R, retrospective; P, prospective; NT-proBNP, N-terminal pro B-type natriuretic peptide; F/U, follow-up; NR, not reported; ED, emergency department; ICU intensive care unit.

**Table-II:** Risk of bias in included studies.

Study	Selection of participants	Confounding variables	Measurement of exposure	Blinding of outcome assessment	Incomplete outcome data	Selective outcome reporting
Jin 2024	Low risk	Unclear risk	Low risk	High risk	Low risk	Unclear risk
Timilsina 2023	Low risk	Unclear risk	Low risk	High risk	Low risk	Unclear risk
Hong 2023	Low risk	Low risk	Low risk	High risk	Low risk	Unclear risk
Datta 2021	Low risk	Low risk	Low risk	High risk	Low risk	Unclear risk
Aarsetoy 2020	Low risk	Low risk	Low risk	High risk	Low risk	Unclear risk
Myhre 2016	Low risk	Low risk	Low risk	High risk	Low risk	Unclear risk
Huang 2016	Low risk	Low risk	Low risk	High risk	Low risk	Unclear risk
Frydland 2016	Low risk	Low risk	Low risk	High risk	Low risk	Unclear risk
Platek 2015	Low risk	High risk	Low risk	High risk	High risk	Unclear risk
Sodeck 2007	Low risk	Low risk	Low risk	High risk	Low risk	Unclear risk
Nagao 2004	Low risk	High risk	Low risk	High risk	Low risk	Unclear risk

### BNP and cardiac arrest outcomes:

**Fig.2:**
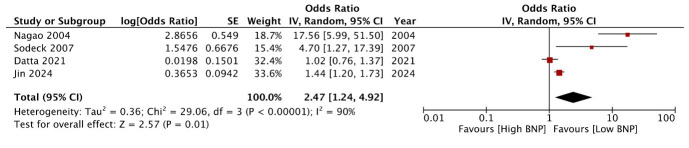
Meta-analysis of the association between BNP and mortality after cardiac arrest.

Four studies^11^,^17^,^23^,^24^ reported data on the relationship between baseline BNP levels and mortality after cardiac arrest. Three^11^,^23^,^24^ of the four studies identified a statistically significant link between elevated BNP levels and mortality following cardiac arrest, while only the study by Dutta et al.^17^ revealed no statistically significant association. The study of Nagao et al.^24^ was an outlier, as it reported only crude data and included patients without ROSC. The remaining three^11^,^17^,^23^ studies reported multivariate adjusted data and included only those patients who had ROSC. Meta-analysis of all four studies^11^,^17^,^23^,^24^ showed a statistically significant association between high baseline BNP levels and mortality after cardiac arrest (OR: 2.47 95% CI: 1.24, 4.92 I^2^=90%) (Fig.2). However, on exclusion of the study of Nagao et al.^24^ the results turned non-significant (OR: 1.37 95% CI: 0.91, 2.07 I²=73%), indicating no relationship between BNP and mortality in resuscitated cardiac arrest patients. Likewise, the results turned non-significant on exclusion of Dutta et al.^17^ and Jin et al.^11^

Two studies^13^,^24^ reported an association between baseline BNP and ROSC. ROSC, defined as sustained ROSC that lasted more than 20 min, was found to be significantly associated with high baseline BNP levels in the study of Hong et al.^13^ The authors showed that patients with the highest quartile of BNP (>426.9 pg/mL) had significantly higher odds of ROSC compared to those with the lowest quartile (<48.88 pg/mL) after adjusting for multiple confounders (OR: 2.375 95% CI: 1.383, 4.078). On the other hand, Nagao et al.^24^ found that there was no statistically significant difference in the number of patients achieving ROSC (defined as return of circulation for at least one minute) between the highest BNP quartiles (>392.1 pg/mL) (31/101) vs the lowest BNP quartiles (<33.8 pg/mL) (46/100) (p=0.1).

The same two studies^13^,^24^ also reported data on survival to discharge and both found opposing results. Hong et al.^13^ in a multivariate adjusted analysis found no significant association between BNP and survival to discharge in cardiac arrest patients (OR: 0.608 95% CI: 0.264, 1.402). However, Nagao et al.^24^ found that baseline BNP was a strong independent predictor of survival to discharge in cardiac arrest patients (OR: 13 95% CI: 4.4, 38.3).

**Fig.3:**
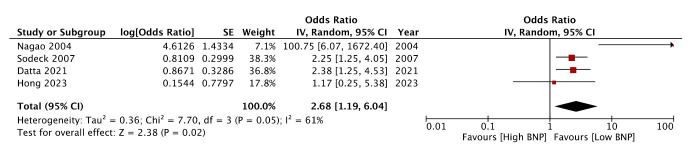
Meta-analysis of the association between BNP and poor neurological outcomes after cardiac arrest.

Four studies^13^,^17^,^23^,^24^ reported an association between BNP and poor neurological outcomes. Only Hong et al.^13^ noted no significant association between BNP and neurological outcomes while the remaining three studies found a significant link between the two. All studies defined poor neurological outcome as a cerebral-performance category score of >2. Pooled analysis of data indicated that high baseline BNP levels were predictive of poor neurological outcomes (OR: 2.68 95% CI: 1.19, 6.04 I^2^=61%) (Fig.3). Again, the study of Nagao et al.^24^ was found to be an outlier, showing a large effect size on the forest plot. However, exclusion of the study did not change the significance of the results (OR: 2.19 95% CI: 1.44, 3.33 I^2^=0%). However, during sensitivity analysis the results turned non-significant on exclusion of Sodeck et al.^23^ and Dutta et al.^17^

### NT-proBNP and cardiac arrest outcomes:

**Fig.4:**
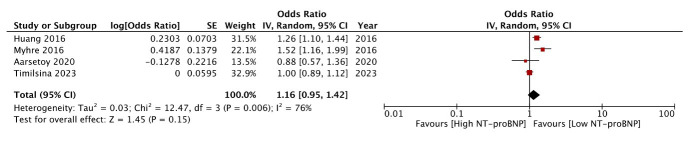
Meta-analysis of the association between NT-proBNP and mortality after cardiac arrest.

Four studies^12^,^18^-^20^ reported analyzable data on mortality, of which two studies found a significant association between baseline NT-proBNP and mortality, while another two found no significant relation. Timilsina et al.^12^ found that there was no association between baseline NT-proBNP and in-hospital (OR: 1.0 95% CI: 0.89, 1.09) or 7-day mortality (OR: 1.0 95% CI: 0.89, 1.09). Aarsetoy et al.^18^ also showed that high baseline NT-proBNP levels did not predict mortality in cardiac arrest patients (OR: 0.88 95% CI: 0.57, 1.36). Similar non-significant results were noted on a subgroup analysis of patients surviving hospital admission (OR: 0.79 95% CI: 0.41, 1.49). On the other hand, Myhre et al.^19^ and Huang et al.^20^ both found baseline NT-proBNP to be a significant predictor of mortality. Pooled analysis showed that NT-proBNP was not a significant predictor of mortality after cardiac arrest (OR: 1.16 95% CI: 0.95, 1.42 I^2^=76%) (Fig.4). No change in the significance of results was noted on sensitivity analysis. Additionally, two other studies also examined mortality based on NT-proBNP levels but did not report analysable data. According to Fryland et al.^21^ survivors had higher levels of NT-proBNP than non-survivors, and Kaplan-Meier curves showed that the likelihood of survival dramatically dropped with increasing quartiles of NT-proBNP. Similarly, Platek et al.^22^ also found that survivors had higher levels of NT-proBNP as compared to non-survivors but the authors did not conduct a multivariate analysis to examine if the biomarker was an independent predictor of mortality.

Only Timilsina et al.^12^ reported the association between NT-proBNP, ROSC and survival to discharge. While the authors noted that baseline NT-proBNP levels were significantly lower in the ROSC as compared to the no-ROSC group, adjusted analysis showed that baseline NT-proBNP levels did not predict ROSC (OR: 1.0 95% CI: 0.89, 1.09). The study also showed no difference in NT-proBNP levels between patients surviving and not surviving to discharge, with multivariate analysis confirming the results.

Association between NT-proBNP and neurological outcomes was reported by two studies. Both studies^19^,^21^ defined poor neurological outcome as cerebral-performance category score of >2. Both Myhre et al.^19^ (OR: 1.5 95% CI: 1.15, 1.95) and Frydland et al.^21^ (OR: 1.8 95% CI: 1.18, 2.76) found NT-proBNP to be a significant predictor of poor neurological outcomes. Frydland et al.^21^ also assessed poor neurological outcomes based on the modified ranking scale (score of 4-6) and found NT-proBNP to be an independent predictor of the same (OR: 2.03 95% CI: 1.32, 3.12).

## DISCUSSION

Biomarkers have recently emerged as essential diagnostic, risk stratification, and treatment decision-making tools in cardiovascular disorders. At present, numerous promising novel biomarkers are under research.^4^,^5^ Nevertheless, the majority of these biomarkers are not appropriate for therapeutic use,^25^ except for cardiac troponins and natriuretic peptides (BNP and NT-proBNP) which are essential for the diagnostic evaluation of patients with acute coronary syndromes and heart failure, respectively.^10^,^26^ These markers have demonstrated their diagnostic efficacy in several investigations and have consequently advanced from laboratory research to clinical application. However, a similar marker is yet to be identified for cardiac arrest. Given that BNP and NT-proBNP have demonstrated value in predicting outcomes of heart failure, we reviewed evidence on their ability to predict outcomes after cardiac arrest.

At the outset, it can be stated that the evidence on the prognostic ability of BNP and NT-proBNP for cardiac is limited and conflicting. Our meta-analysis, after excluding an outlier study,^24^ noted no significant relationship between BNP and mortality. Results of individual studies were also conflicting. Dutta et al.^17^ did not note any significant association with mortality while three other studies^11^,^23^,^24^ found BNP to be a predictor of mortality. Studies reporting positive association adjusted for important confounders like age, diabetes, chronic heart failure, initial rhythm, cumulative adrenaline administration, time to ROSC, and initial lactate levels while Dutta et al.^17^ did not clearly mention the factors adjusted for mortality. Other possible factors leading to variable results may include, time of the study, follow-up, aetiology of cardiac arrest and different BNP cut-offs. Due to the scarce data, we could not perform subgroup or meta-regression analysis to decipher the effect of these variables on the outcomes. The meta-analysis also provided evidence that BNP could be an independent predictor of neurological outcomes. All studies used a consistent definition of poor neurological outcomes and even after exclusion of an outlier study, the results indicated a two-fold risk of worse neurological outcomes in those with high BNP levels.

Likewise, contrasting results were noted for studies examining the prognostic role of NT-proBNP and mortality. Descriptive analysis revealed that some studies noted a positive association between NT-proBNP and mortality or higher levels in survivors, however, the meta-analysis failed to show that NT-proBNP was of value in predicting mortality after cardiac arrest. The strongest association was noted by the study of Myhre et al.^19^ which showed a 50% increase in the risk of mortality with elevated NT-proBNP levels which could be due to the longer follow-up of the study (One Year). On the other hand, two studies reporting the relationship between of NT-proBNP and neurological outcomes concurred on the prognostic ability of the marker indicating a 50-80% higher risk of worse neurological outcomes.

It’s unclear exactly how BNP and NT-proBNP functions as a mediator or marker of poor neurological function. Numerous investigations have established their prognostic significance in patients with different neurological disorders.^27^-^30^ Ru et al.^27^ have shown that both markers are increased in the cerebrospinal fluid of individuals with traumatic brain injury and this may be indicative of their origin from the brain. A meta-analysis of 16 studies shows that both markers can predict post-stroke mortality.^28^ Lu et al.^30^ have shown that BNP is predictive of functional outcomes in non-cardiogenic stroke while another study^29^ demonstrates the NT-proBNP can predict both haemorrhagic transformation and functional outcomes after acute stroke. Patients with subarachnoid hemorrhage who have elevated BNP levels have shown delayed recovery of cerebral function.^31^ Natriuretic peptides appear to be extensively distributed throughout the central nervous system, indicating their potential involvement in regulating physiological activities.^32^ It is plausible that their exists unexplored mechanisms by which these natriuretic peptides affect neurological outcomes and only further basic research can provide valuable insights.

There also exists contrasting explanations regarding the prognostic role of BNP/NT-proBNP in cardiac arrest. On one hand, it is suggested that cardiac arrest induces ischemia and hypoxia in the body, resulting in the transcription of NPPB genes and, consequently, an elevation in BNP and NT-proBNP production. The immunological inflammatory response triggered by ischemia/reperfusion can also elevate production natriuretic peptides.^11^,^20^ On the other hand, BNP/NT-proBNP is a dynamic metric that fluctuates considerably with changes in loading conditions.^17^ Baseline conditions like atrial fibrillation and myocardial ischemia can be associated with elevated BNP/NT-proBNP levels and hence results can be confounded by these variables.

However, Jin et al.^11^ have shown that BNP remains persistently high even in survivors with such cardiac conditions as compared to non-survivors. The exact timing of sample collection and initial rhythm could also affect outcomes as Datta et al.^17^ have noted that patients exhibiting asystole or pulseless electrical activity as their first rhythm demonstrated a greater likelihood of elevated BNP levels. It is also important to mention that the cause of cardiac arrest can influence the levels of natriuretic peptides since they are generated by division of pro-BNP secreted by the cardiomyocytes.^33^ About 63% of cardiac arrests are caused by hypertrophic cardiomyopathy, myocarditis, and abnormalities of the coronary arteries.^22^

However, not all cardiac arrests originate from cardiac causes. It has been demonstrated that in only 39% of IHCA patients, the initial cause was cardiac. Non-cardiac aetiologies of cardiac arrest encompass bleeding, intracranial conditions, pulmonary disease, cancer, intoxication, and trauma.^34^ In this context, it is beneficial to acknowledge that cardiac arrest patients constitute a heterogeneous cohort, whose early attributes affect the outcome. Moreover, time is crucial at every step of the process, from initial diagnosis to administration of treatment. Furthermore, time is critical at every step of the process, from diagnosis to administration of treatment, and in such emergency settings, it is difficult to perform meaningful research by precisely classifying patients based on etiology and treatment procedures. Therefore, despite numerous studies, there will exists variations among patients and the value of these biomarkers.

### Limitations:

Firstly, this study was more of a systematic review than a meta-analysis as small number of studies precluded a detailed analysis of all outcomes. Most data was regarding mortality and neurological outcomes and few studies reported on ROSC and survival to discharge. The interpretation of these findings should also acknowledge that most of the evidence is observational. Most studies were cohort studies, many of which were retrospective, and all carry a high risk of bias in outcome assessment blinding, with varying levels of confounding control. We also underline that inter-study heterogeneity is a significant issue that has resulted in inconsistent outcomes and precludes the therapeutic application of both markers. Several factors, like whether the arrest was witnessed, timing and the quality of bystander cardiopulmonary resuscitation, the duration of the ventricular fibrillation, the duration of resuscitation, timing of defibrillation, administration of adrenaline, survival to hospital admission, comorbidities, aetiology of arrest, location of arrest, etc. can be confounders affecting outcomes. A thorough analysis including all of these factors was not conducted by any study due to lack of adequate data collection given the emergency nature of the incident. Moreover, inconsistent and often incomplete adjustment for key confounders across included studies may have introduced residual confounding, which may partly explain the observed heterogeneity and limit the reliability and external validity of the pooled estimates.

A key source of variability in this review stems from how and when ’baseline” BNP and NT-proBNP levels are measured. Different studies collected blood samples at various times, such as during CPR, upon emergency department arrival, after spontaneous circulation returned, at ICU admission, or several hours post-cardiac arrest. Since natriuretic peptides are highly dynamic—affected by factors like myocardial ischemia–reperfusion injury, hemodynamic instability, volume status, and catecholamines—measurements taken during different resuscitation phases likely reflect distinct underlying processes. Pooling these data could mask how prognostic significance changes over time and may contribute to the notable heterogeneity across studies. We were unable to perform a detailed subgroup analysis for all confounding variables due to the limited number of studies in each meta-analysis. Data was also from varying locations with differing quality of emergency medical services. There were also inconsistencies among studies regarding the reporting of data with some comparing only values of the markers between survivors and non-survivors limiting their inclusion in the meta-analysis. Several of the studies were also retrospective in nature and a large number of patients had to be excluded as data on the markers was missing.

## CONCLUSION

Evidence on the prognostic ability of BNP and NT-proBNP for cardiac arrest is preliminary. BNP and NT-proBNP may potentially predict poor neurological outcomes after cardiac arrest. Limited and inconsistent evidence also shows that there may be no association between baseline level of these markers and mortality. Data on ROSC and survival to discharge is scarce and conflicting and needs more studies for robust conclusions.

### Authors’ contributions:

**YZ and JW:** Literature search, study design and manuscript writing.

**YQ and ZZ:** Data collection, data analysis and interpretation. Critical Review.

**YZ and JW:** Manuscript revision and validation and is responsible for the integrity of the study.

All authors have read and approved the final manuscript.
